# Parallel Corpus Analysis of Text and Audio Comprehension to Evaluate Readability Formula Effectiveness: Quantitative Analysis

**DOI:** 10.2196/69772

**Published:** 2025-10-02

**Authors:** Arif Ahmed, Gondy Leroy, David Kauchak, Prosanta Barai, Philip Harber, Stephen Rains

**Affiliations:** 1 Management Information Systems (MIS), Eller College of Management, The University of Arizona, 401 E University Blvd, Tucson, AZ, 85719, United States, 1 (520) 621-2211; 2 Computer Science Department, Pomona College, Claremont, CA, United States; 3 Department of Public Health, University of Arizona, , Tucson, AZ, United States; 4 Department of Communication, University of Arizona, , Tucson, AZ, United States

**Keywords:** health literacy, parallel corpora, generative AI, user evaluation, text and audio readability, comprehension, retention, actual difficulty, perceived difficulty.

## Abstract

**Background:**

Health literacy, the ability to understand and act on health information, is critical for patient outcomes and health care system effectiveness. While plain language guidelines enhance text-based communication, audio-based health information remains underexplored, despite the growing use of digital assistants and smart devices in health care. Traditional readability formulas, such as Flesch-Kincaid, provide limited insights into the complexity of health-related texts and fail to address challenges specific to audio formats. Factors like syntax and semantic features significantly influence comprehension and retention across modalities.

**Objective:**

This study investigates features that affect comprehension of medical information delivered via text or audio formats. We also examine existing readability formulas and their correlation with perceived and actual difficulty of health information for both modalities.

**Method:**

We developed a parallel corpus of health-related information that differed in delivery format: text or audio. We used text from the British Medical Journal (BMJ) Lay Summary (n=193), WebMD (n=40), Patient Instruction (n=40), Simple Wikipedia (n=243), and *BMJ* journal (n=200). Participants (n=487) read or listened to a health text and then completed a questionnaire evaluating perceived difficulty of the text, measured using a 5-point Likert scale, and actual difficulty measured using multiple-choice and true-false questions (comprehension) as well as free recall of information (retention). Questions were generated by generative artificial intelligence (ChatGPT-4.0). Underlying syntactic, semantic, and domain-specific features, as well as common readability formulas, were evaluated for their relation to information difficulty.

**Results:**

Text versions were perceived as easier than audio, with BMJ Lay Summary scoring 1.76 versus 2.1 and *BMJ* journal 2.59 versus 2.83 (lower is easier). Comprehension accuracy was higher for text across all sources (eg, *BMJ* journal: 76% vs 58%; Patient Instructions: 86% vs 66%). Retention was better for text, with significant differences in exact word matching for Patient Instructions and *BMJ* journal. Longer texts increased perceived difficulty in text but reduced free recall in both modalities (−0.23,−0.25 in audio). Higher content word frequency improved retention (0.23, 0.21) and lowered perceived difficulty (−0.20 in audio). Verb-heavy content eased comprehension (−0.29 in audio), while nouns and adjectives increased difficulty (0.20, 0.18). Readability formulas’ outcomes were unrelated to comprehension or retention, but correlated with perceived difficulty in text (eg, Smog Index: 0.334 correlation).

**Conclusions:**

Text was more effective for conveying complex health information, but audio can be suitable for easier content. In addition, several textual features affect information comprehension and retention for both modalities. Finally, existing readability formulas did not explain actual difficulty. This study highlighted the importance of tailoring health information delivery to content complexity by using appropriate style and modality.

## Introduction

### Background

Clear and understandable information is vital in health care to enhance health literacy. Improving health literacy is a significant national goal with numerous benefits [1] such as making better-informed choices, managing chronic illnesses, averting health issues, and reducing health care expenses [2,3]. The US National Action Plan aims to improve health literacy, emphasizing the need for clear health-related information, while the Plain Writing Act promotes clarity in communication [4]. Although there are many plain language guidelines for delivering health information through text, audio is often ignored. Incorporating audio guidelines for providing health information could offer a substantial opportunity to improve health literacy [5,6].

Audio formats are gaining popularity as mobile devices and smart speakers with digital assistants (eg, Siri and Alexa) become increasingly prevalent for accessing information. In 2022, approximately 142 million people in the United States used digital assistants, representing nearly half of the population. By 2026, this number is projected to rise to 157.1 million users [7]. These devices are also incorporated into health care settings, allowing patients to converse with medical professionals and seek information [8]. Patients can pose health-related questions and receive responses, provided the information is clear and understandable. In 2019, health-related inquiries accounted for 16% of all smart speaker interactions [9]. American adults’ utilization of digital assistants for health care queries experienced a notable increase, surging from 19 million in 2019 to 51.3 million in 2020 and further to 54.4 million in 2021 [10].

There are currently no established metrics for measuring the difficulty of health information conveyed through audio [11]. Yet, text difficulty metrics such as the Flesch-Kincaid Grade Level, Gunning Fog Index, and Dale-Chall Readability Score are prevalent. These metrics typically consider factors such as sentence length, word frequency, and syllable count to estimate the reading level or educational grade required to understand a given text. While these metrics provide useful approximations of text complexity, they have limitations and fail to account for factors like background knowledge or syntactic or semantic features of texts [12].

### Health Literacy/Plain Language

Health literacy is crucial to effective health care communication and patient education. Health literacy refers to an individual’s ability to obtain, process, and comprehend basic health information and services necessary to make appropriate health decisions. This concept extends beyond the ability to read and write, encompassing skills such as numeracy, decision-making, and critical thinking in health contexts [13]. The importance of health literacy cannot be overstated, as it has a direct impact on patient outcomes and health care system efficiency. Individuals with low health literacy often struggle to understand medical instructions, manage chronic conditions, and navigate complex health care systems [14]. This can lead to increased hospitalization rates, poor medication adherence, and higher health care costs. Recognizing this, health care providers and policymakers have increasingly focused on improving health literacy through various initiatives [15]. Plain language is a key strategy in addressing health literacy challenges. It involves using clear, concise, and jargon-free communication to convey complex health information. The principles of current plain language advice include using everyday words, short sentences, active voice, and logical organization of information [16]. By implementing plain language practices, health care providers can significantly enhance patient understanding, resulting in improved health outcomes and increased patient satisfaction [17].

### Difficulty Measures

The readability of source texts is another critical factor in health communication. Readability refers to the ease with which a reader can understand written text. In health care, ensuring that patient education materials, consent forms, and medical instructions are easily readable is paramount. Various factors, including vocabulary complexity, sentence length, and overall text structure, influence readability [18]. Readability formulas, such as Flesch-Kincaid, are commonly used to evaluate text readability [19]. However, research indicates that Flesch-Kincaid is ineffective in evaluating text simplification metrics [20].

Recent endeavors to simplify text have concentrated on semantic and syntactic analysis to identify features that mitigate text difficulty. Qualitative elements such as linguistic conventions, clarity, depth of content, and the reader’s background knowledge significantly impact perceived difficulty. Quantitative measures, such as word frequency, grammar frequency, sentence length, and sentence structure, also impact understanding [21]. Word frequency measures how often specific words appear in everyday language and can be measured with resources such as the Google web corpus [21]. Grammar frequency refers to how common high-level grammatical structures are used in language. Sentences with more familiar structures are easier to read and process, contributing to better overall comprehension [22]. For example, texts that are harder to read often have low word frequency, fewer verbs and function words, more nouns, and more complex vocabulary [23]. Lexical chains (ie, topics in text), their length, and how they are intertwined can also differentiate between difficult and easy texts [24]. Metrics such as specificity, ambiguity, concept density, and topic density are used to determine the difficulty of medical texts [25]. Advanced tools like Coh-Metrix analyze multiple dimensions of text, including cohesion, syntactic complexity, and word concreteness. These analyses provide a more nuanced understanding of what makes a text challenging. Such measures are particularly valuable in educational settings and in developing materials for diverse audiences [26].

Although research focusing specifically on delivering information in audio format is limited, there is evidence that features like audio speech rate, pauses, and emphasis can play an important role in the comprehension of audio content [27,28]. The Speech Transmission Index and Speech Intelligibility Index are examples of tools used to evaluate the clarity of spoken content [29]. Further research is needed to explore how text and audio features affect the difficulty of health-related information [30]. Audio difficulty measures are important, especially in an era where health information is increasingly disseminated through audio formats via digital assistants. In health care settings, audio difficulty measures are needed to ensure that verbal instructions, telemedicine consultations, and health-related audio content are accessible to all patients, including those with hearing impairments or nonnative speakers. By considering both text and audio difficulty, health care providers can create more inclusive and effective communication strategies. In this study, we evaluate user comprehension and retention of information delivered via text and audio. We also examine the features of the texts that determine the comprehensiveness of the information in both modalities and assess the effectiveness of existing readability formulas in evaluating the difficulty of the information. Our goal is to analyze how health information is comprehended when delivered via text versus audio. We aim to evaluate which method is more effective in facilitating the understanding and retention of the information. In addition to this comparison, we investigate the features of the underlying text and its effect in each modality to determine their respective strengths and weaknesses in conveying health-related content.

## Methods

### Corpus Creation

We developed a parallel corpus of health-related texts using common diseases and health conditions listed in the *ICD-10* (*International Statistical Classification of Diseases, Tenth Revision*) coding system [31]. To ensure a representative and diverse corpus of health information, we selected texts from 5 sources that span a broad spectrum of difficulty, style, and intended audience: *British Medical Journal* (*BMJ*; 200 texts), BMJ Lay Summary (193 texts), WebMD (40 texts), Patient Instruction (40 texts), and Simple Wikipedia (243 texts). These were chosen to create a diverse corpus. WebMD and Patient Instruction have fewer texts since they did not contain text for all diseases in our list.

The *BMJ* is a peer-reviewed medical journal that publishes highly technical, scientific content written by medical experts and researchers. The language is largely technical, with extensive use of medical terminology and detailed scientific information. The tone is formal and objective, adhering to strict academic standards. In contrast, BMJ Lay Summaries present the key findings and implications in a more accessible, easy-to-understand manner for a general audience. The language is largely nontechnical, the tone is more friendly and engaging, and the structure is more narrative-driven. WebMD is a popular web-based health resource for a general, nonmedical audience. The articles on WebMD cover a wide range of health-related topics, using relatively simple and straightforward language with minimal technical jargon. The tone is informative and conversational, designed to educate and empower readers to make informed decisions about their health. Patient Instructions are educational materials specifically designed to provide clear, step-by-step guidance to patients on various medical procedures, treatments, or self-care practices. These materials are typically authored by clinical educators or health care providers to guide patients on managing specific conditions, procedures, or treatments. For this study, we sourced materials from publicly accessible sections of US-based hospital and clinic websites that provide downloadable or viewable patient instructions (eg, Children’s Hospital of Philadelphia, University of Rochester Medical Center). The language used is simple, direct, and easy to understand, focusing on providing practical, hands-on information. The tone is friendly and reassuring, and the structure is highly organized, often with numbered or bulleted steps. Simple Wikipedia is a version of the popular online Wikipedia that uses simpler language and explanations to make the content more accessible to a wider audience. The language is straightforward, with shorter sentences, simpler vocabulary, and minimal use of complex terminology or jargon. The tone is informative and objective, and the structure is typically organized with clear headings, subheadings, and bullet points to enhance readability and comprehension.

To create the parallel corpus with the texts presented in audio format, we used Microsoft Azure’s text-to-speech service using the default US male voice and the default speech rate settings. We chose not to manipulate prosodic features such as emphasis or pauses, instead relying on Azure’s default settings to ensure consistency across audio samples. This decision allowed us to isolate the effect of modality (text vs audio) while minimizing confounding variability introduced by TTS customization. Our prior work using the same TTS system showed that the default speech rate produced better comprehension than adjusted rates, and comprehension did not differ significantly by voice gender [27].

### Text Features

We generated information features for each text based on our and other researchers’ findings of important textual elements that have been shown to impact readability, understandability, and retention (Table 1) [22-24,32-34]. Table 1 is intended to establish a comprehensive baseline for understanding how various publicly accessible health-related texts differ in their linguistic and structural complexity. Overall, there are 4 groups of features.

**Table 1.** Text readability features for the corpora.

Features	BMJ^a^ Lay Summary (n=193)	WebMD (n=40)	Patient instructions (n=40)	Simple Wikipedia (n=243)	*BMJ* journal (n=200)
Average word count	205	595	961	353	237.335
Ordinariness					
Content word frequency	364,959,457.80	387,640,06.50	299,361,102.70	498,830,364.50	235767137.50
Grammar frequency	9882.89	5495.91	3108.28	6883.26	4916.36
Health care domain specialty (averages)					
Specificity	0.210	0.384	0.364	0.122	0.220
Ambiguity	0.245	0.433	0.422	0.1557	0.337
Concept density	0.231	0.388	0.341	0.114	0.3472
Topic density	0.411	0.505	0.559	0.329	0.4879
Parts-of-speech features (%)					
Nouns	30.90	23.31	32.35	30.91	34.28
Verbs	16.82	19.51	16.34	17.06	12.605
Adverbs	4.00	5.86	2.87	4.94	3.37
Adjectives	10.34	9.21	7.84	9.60	12.875
Topic spread (averages)					
Lexical chains	0.224	0.396	0.326	0.123	0.2973
Lexical chain length	0.484	0.277	0.354	0.310	0.3911
Lexical chain span	0.195	0.362	0.274	0.113	0.288
Lexical cross chains	0.222	0.405	0.330	0.122	0.2971

a *BMJ: British Medical Journal.*

The first group of features, “Ordinariness,” focuses on text difficulty using word and grammar frequencies [23]. Content word frequency, indicating vocabulary familiarity, is highest for Simple Wikipedia at 498 million, followed by WebMD (387 million), with the *BMJ* journal having the lowest frequency (236 million), which suggests a greater use of jargon language. Grammar frequency is highest in BMJ Lay Summaries (n=9882), while Patient Instructions (n=3108) and *BMJ* journal (n=4916) are lower, reflecting their more syntactical nature.

The second group, “Healthcare Domain Specialty,” comprises specificity, ambiguity, concept density, and topic density. In specialized medical content, these metrics reflect the difficulty of a text based on the complexity of the medical terms it contains. Texts with more specific, ambiguous, or conceptually dense terms are harder to comprehend [25]. Specificity for WebMD and Patient Instructions is high, with values of 0.384 and 0.364, respectively, while Simple Wikipedia has lower specificity (0.122). Ambiguity is highest for WebMD (0.433), followed closely by Patient Instructions (0.422), suggesting more generalized content. *BMJ* journal (0.337) and Simple Wikipedia (0.1557) show the lowest ambiguity. Concept density and topic density are highest for WebMD (0.388 and 0.505, respectively), indicating that the content contains more information, while Simple Wikipedia is the least dense, with values of 0.114 and 0.329.

The third group includes “Parts-Of-Speech Features” where a higher proportion of nouns and adjectives is linked to more difficult text, while more verbs and adverbs make text easier [25]. For parts of speech, the *BMJ* journal has the highest percentage of nouns (34.28%) and adjectives (12.88%), indicating difficult content. WebMD uses more verbs (19.51%), making it easier, while Patient Instructions use the highest percentage of nouns (32.35%) [23]. Adverbs are used more frequently in WebMD (5.86%), while *BMJ* journal has the fewest (3.37%).

The fourth group, “Topic Spread,” focuses on the topics in the text, measured by lexical chains [24]. Lexical chain analysis helps evaluate topic distribution and repetition within the text, with longer chains and fewer overlaps associated with simpler texts [23]. WebMD has the highest lexical chain score (0.396), suggesting greater word repetition and coherence, while BMJ Lay Summaries and Simple Wikipedia have the lowest values (0.224 and 0.123, respectively). Lexical chain length and lexical cross chains also follow a similar pattern, with WebMD showing the highest values across these metrics, while Simple Wikipedia and BMJ Lay Summaries have the lowest, making them more varied and straightforward [24].

### Study Overview and Participant Recruitment

Two parallel studies were conducted for each text source: an audio study to assess auditory information processing and a text study to evaluate textual information processing. The procedure was identical for each study. Participants were presented with either audio or textual health information and then asked to complete a questionnaire to evaluate their perception and understanding of the information.

Study participants were recruited through Amazon Mechanical Turk (AMT). First, we screened AMT workers using standard criteria, including US residency and a 98% approval rating. We then collected demographic information from 1000 workers through a survey, in which we also provided 3 audio snippets containing a word with varied noise levels. The workers who correctly identified at least 2 audio snippets were further invited to participate in the study. This approach enabled us to ensure that all participants had sufficient hearing ability to comprehend the audio information.

Participants received US $0.50 for each completed human intelligence task (HIT). While completing multiple HITs was allowed, participants were prevented from evaluating duplicate content (eg, both the audio and printed versions of a text) to prevent potential bias from repeated exposure. Each text or its audio version represented one HIT containing the stimulus material (either text or audio) followed by a short questionnaire containing a perceived difficulty evaluation, 2 multiple-choice (MC) comprehension questions, 2 true-false (TF) comprehension questions, an attention check question (for data cleaning purposes), and a free recall task.

### Ethical Considerations

This study was reviewed and approved by the Institutional Review Board (IRB) at the University of Arizona (IRB Protocol: STUDY00002235). It involves human participants and complies with all institutional and federal guidelines for the ethical conduct of research. This study involved data collection through AMT, and full IRB approval was obtained before data collection.

Before participation, all participants were provided with an electronic informed consent form. The form outlined the purpose of the study, the procedures involved, and participants’ rights, including the right to withdraw from the study at any point without penalty. Participants were informed that the IRB had approved the study.

All data collected in this study were deidentified before analysis. No personally identifiable information was collected or stored. Data were stored on secure, access-controlled servers, and all analyses were conducted using anonymized datasets to ensure participant confidentiality.

Participants were compensated for their time through the AMT platform. Each participant received US $0.50 for completing a HIT, including reading materials and answering questions. No images or other materials containing identifiable participant information were used in the manuscript or supplementary materials.

### Measures

Our study used a multifaceted approach to assess information comprehension: perceived difficulty, comprehension (actual difficulty), and information retention. To measure perceived difficulty, participants evaluated the audio information using a 5-point Likert scale, ranging from 1=“very easy” to 5=“very difficult.”

To measure comprehension (actual difficulty), we used questions designed to evaluate participants’ understanding of the text. Two MC questions and 2 TF questions were developed for each text using generative artificial intelligence (ChatGPT-4.0). All questions and answers were manually evaluated by a domain expert (ie, a medical doctor) to ensure that they were relevant and appropriate.

To evaluate information retention, participants were asked to recall as much information as possible from the presented information. Participants were presented with a text box and instructed to type everything they could remember about the text. To automatically and objectively assess participants’ information recall, we applied 2 complementary methods: exact word matching and semantic similarity using word embeddings. The exact word match percentage was computed by preprocessing participant responses and the source text (eg, lowercasing, removing punctuation, and stop words) and calculating the proportion of words in the recall that appeared verbatim in the original text. This method provides a straightforward measure of literal recall but may fail to capture meaning when participants paraphrase. To address this limitation, we incorporated a semantic similarity measure based on word2vec embeddings [35]. Word2vec is a neural model that learns distributed word representations from large corpora, positioning semantically similar words closer in a high-dimensional vector space. We calculated the cosine similarity between each word in a participant’s response and words in the original text, averaging the most similar pairs to generate an overall semantic similarity score. This approach enables the detection of meaning-level recall, even when participants use different words than those in the original material. Together, these measures allowed for a more comprehensive evaluation of both literal and conceptual memory performance.

Finally, we included attention-check questions. Participants were asked to identify the most frequently occurring word from a list based on the information provided. The responses to the attention check questions helped us evaluate each HIT response.

## Results

### Data Collection

To ensure better quality data and a reliable evaluation of our study result, we only included answers from workers who passed a quality control process established in previous work that prohibits copy-pasting and incoherent content [36]. HTML-based tools are used to spellcheck and flag errors or nonsensical entries, prompting workers to improve their responses for clarity and professionalism. We followed 3 filtering criteria to create 2 datasets: strict and lenient. First, we reviewed the correct response to the attention check question. If the response was correct, we included the HIT in the strict dataset. If the response was incorrect, the HIT was categorized under the lenient dataset. Then, we removed HITs from the entire dataset whose average accuracy on MC and TF questions was below 25%, indicating random guessing. And, finally, we verified free recall responses as appropriate or not. If it was not appropriate, we removed that HIT from the entire dataset.

In this results section, we provide a detailed description of the results for the strict dataset. Because the results are very similar to those of the lenient dataset, the details of the lenient and strict dataset and data cleaning steps are provided in Multimedia Appendix 1. Representative examples of the text information that were presented to study participants are provided in Multimedia Appendix 1.

For the AMT workers whose data was retained for the analysis, participants were divided into 2 groups based on their interaction with either audio or text. There were 274 participants in the text condition and 213 participants in the audio condition.

Both conditions had a similar distribution of males (≈67%) and females (≈32%; Table S1 in Multimedia Appendix 1). Most participants were between 30 and 39 years old (48%, 103/213, in the audio condition and 55%, 151/274, in the text condition). Approximately one-third of the participants were younger than 30 years, and a small percentage were 40 years or older. The racial makeup was predominantly White (more than 94% in both groups), and 28.16% (60/213) and 26.27% (72/274) identified as Hispanic or Latino in the audio and text conditions, respectively.

Most participants had a bachelor’s degree. A smaller percentage had a master’s degree, and no participants held a doctorate. English proficiency was strong, with over 79% of participants in both conditions reporting that they spoke only English at home.

### Perceived Difficulty

**Figure 1.**
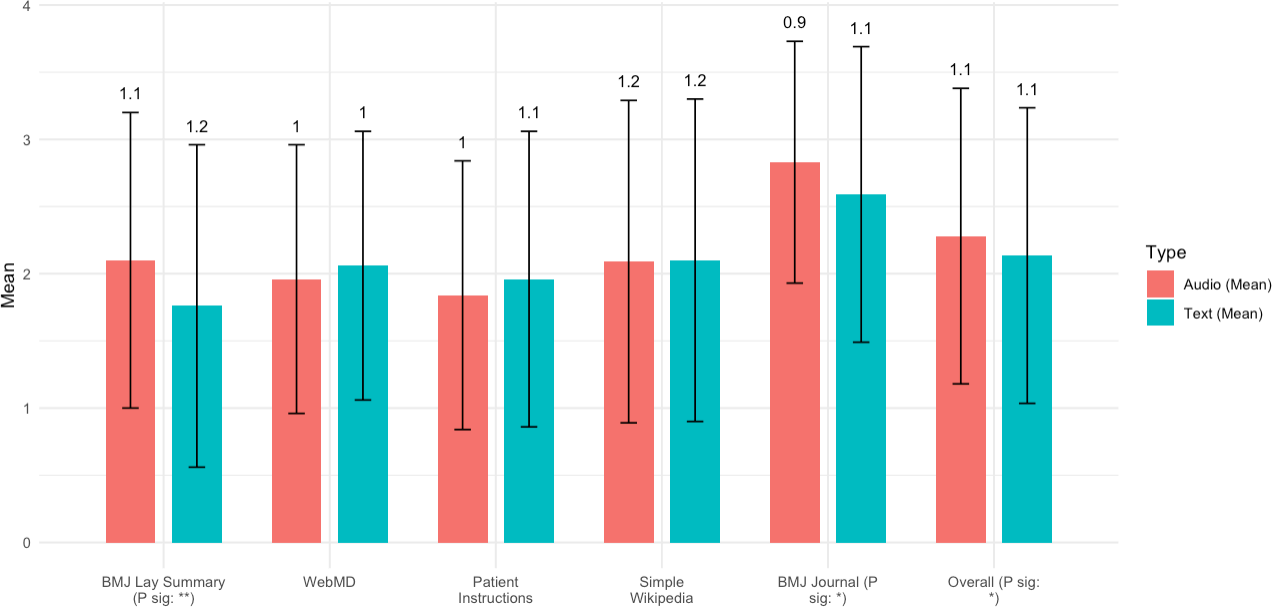
Perceived difficulty results by each source. (SD shown as error bar). *The correlation is significant at a significance level of 0.05 (2-tailed). **The correlation is significant at a significance level of 0.0001 (2-tailed).

Overall, information was perceived as easier to understand when presented as text compared with audio. Our *t* tests (Figure 1) show that the difference is statistically significant for BMJ Lay Summary and *BMJ* journal. A higher perceived difficulty score means the content is difficult to understand. For BMJ Lay summary, text achieves a better perceived difficulty score (1.76) compared with audio (2.1), and for the original *BMJ* journal, the text achieves a better score (2.59) compared with audio (2.83). In contrast, for the WebMD and Patient Instruction sources, which contain easier-to-understand content, the differences are not statistically significant.

### Actual Difficulty: Comprehension and Retention

#### Information Comprehension

**Figure 2.**
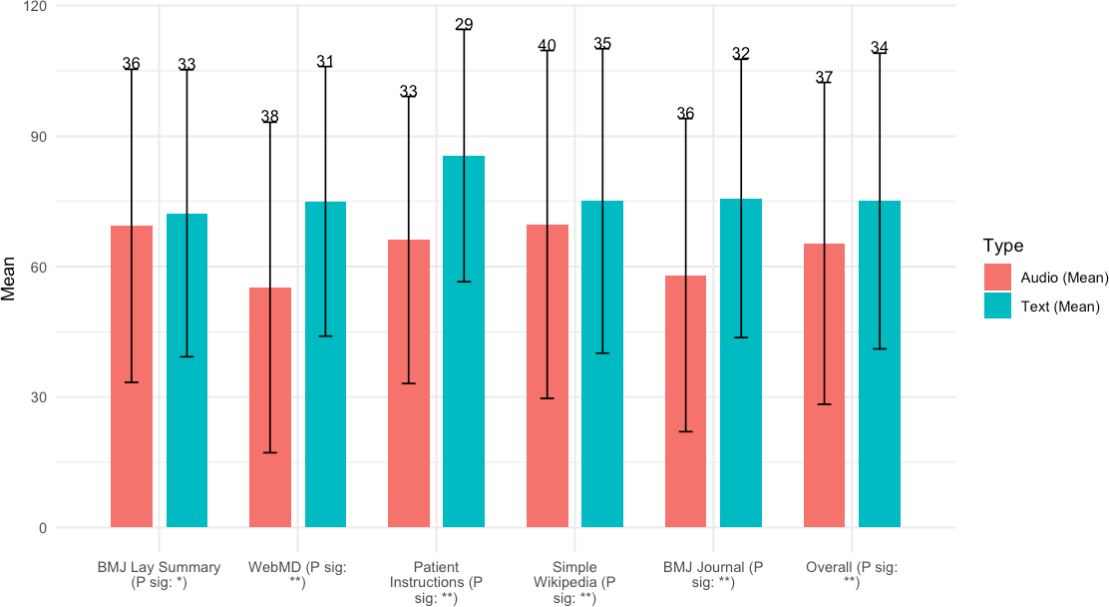
Accuracy (%) for MC and TF questions (SD shown as error bar). *The correlation is significant at a significance level of 0.01 (2-tailed). **The correlation is significant at a significance level of 0.001 (2-tailed).

Figure 2 shows the average accuracy of answering the MC and TF questions. A higher value means greater comprehension. Text consistently yields higher comprehension performance (ie, questions answered correctly) than audio. The difference is statistically significant for all sources. For example, in the BMJ Lay Summary, text achieves 72% accuracy compared with audio’s 69%, while WebMD shows a more pronounced difference, with text reaching 75% and audio trailing at 55%. The largest gap is observed in Patient Instructions, where text comprehension reached 86% versus audio’s 66%. Similarly, for *BMJ* journal, which features more difficult content, text achieves 76%, significantly higher than audio’s 58%. These results highlight that text is more effective than audio in facilitating comprehension, especially for difficult health information.

#### Information Retention

**Figure 3.**
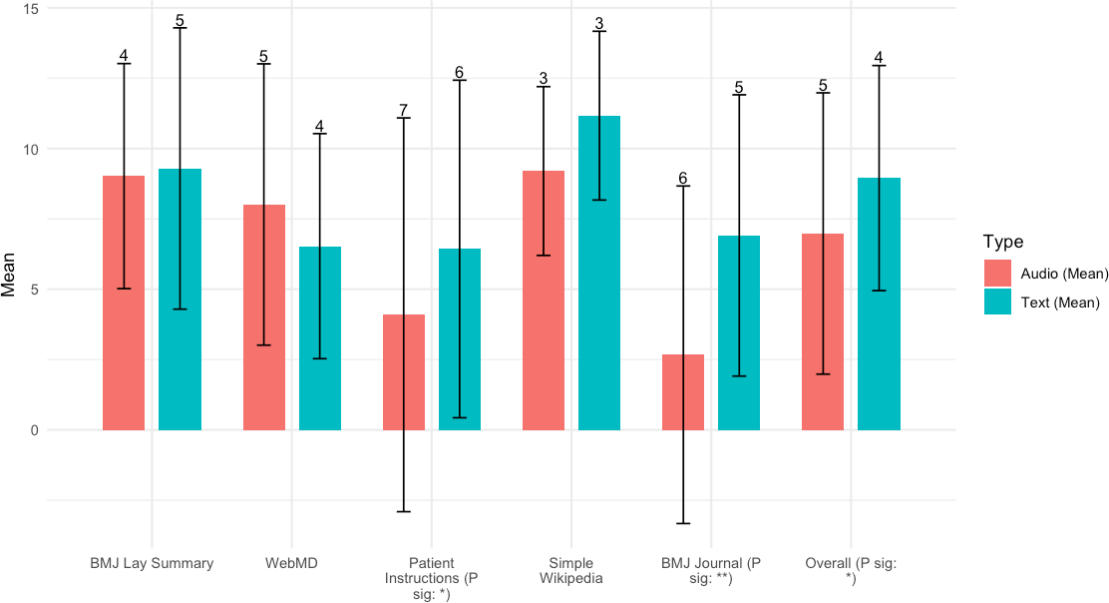
Exact matching words (%) for free recall. (SD shown as error bar). *The correlation is significant at a significance level of 0.05 (2-tailed). **The correlation is significant at a significance level of 0.001 (2-tailed).

Overall, we found that information retention measured using exact word matching (Figure 3) outperforms information retention with text versus audio information. A higher value means better retention. Overall, the result is significant, and text outperforms audio. However, the difference is only significant for Patient Instructions and *BMJ* journal.

**Figure 4.**
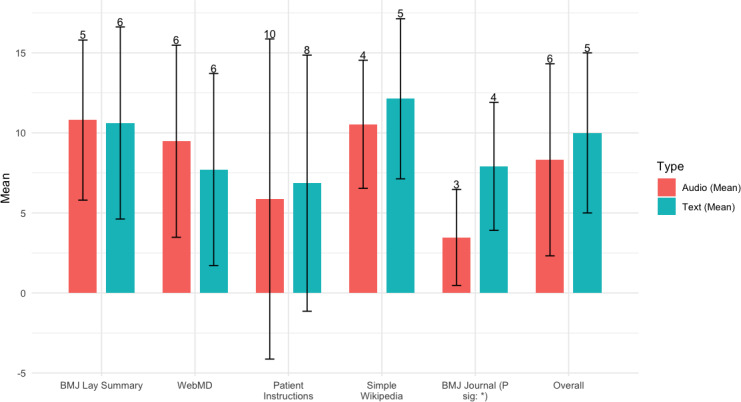
Similar word (%) for free recall. (SD shown as error bar). *The correlation is significant at a significance level of 0.001 (2-tailed).

When using similar words to measure information retention (Figure 4), which allows for more flexibility in recall accuracy, the differences are similar but smaller. A higher value means better retention. There is only a significant difference for the *BMJ* journal. Text still outperforms audio except for WebMD.

### Content Feature Analysis

We analyzed the content features of texts and the correlation with perceived and actual difficulty measures for both modalities. Tables2  3 show the 4 groups of content features and their correlation with text or audio comprehension and retention. For the text condition (Table 2), the first group of features, “Ordinaries” reveals that higher content word frequency correlates with lower perceived difficulty (−0.27) and better free recall (0.23 and 0.21). Grammar frequency has a smaller negative effect on perceived difficulty (−0.14) and a negligible impact on recall (0.02 and 0.013). The second group of features, “Healthcare Domain Specialty,” shows that specificity increases perceived difficulty (0.13) while reducing free recall (−0.22 and −0.23). Ambiguity, concept density, and topic density increase perceived difficulty (0.31, 0.32, 0.33, respectively) and negatively impact free recall (−0.31,−0.32, −0.42, respectively), making densely packed and ambiguous texts difficult to process and remember. The third group of features, “Parts-of-Speech Features,” indicates that a higher percentage of nouns correlates with greater perceived difficulty (0.20), while verbs reduce perceived difficulty (−0.28). Adverbs slightly decrease difficulty (−0.08), but none of these features significantly affect recall. The fourth group of features, “Topic Spread,” shows that texts with more lexical chains are perceived as more difficult (0.21) and result in lower free recall (−0.26 and −0.27). Longer chains further increase perceived difficulty (0.10) and reduce information retention (−0.34 and −0.36). Overlapping topics, indicated by lexical cross chains, follow the same pattern, increasing perceived difficulty (0.21) and decreasing recall (−0.26 and −0.27).

**Table 2.** Correlation of text features with the dependent variables for the strict dataset (information presented as text).

Features	Perceived difficulty	Actual difficulty		
		Comprehension	Information retention	
		MC^a^ and TF^b^	Percentage of exact matching words	Percentage of similar words
Average word count	0.17^c^	0.01	−0.25^c^	−0.25^c^
Ordinariness				
Content word frequency	−0.27^c^	0.03	0.23^c^	0.21^c^
Grammar frequency	−0.14^c^	0.04	0.02	0.013
Health care domain specialty (averages)				
Specificity	0.13^c^	0.01	−0.22^c^	−0.23^c^
Ambiguity	0.31^c^	−0.01	−0.31^c^	−0.31^c^
Concept density	0.32^c^	−0.01	−0.32^c^	−0.32^c^
Topic density	0.33^c^	−0.03	−0.42^c^	−0.42^c^
Parts-of-speech features (%)				
Nouns	0.20^c^	−0.04	0.01	0.01
Verbs	−0.28^c^	0.11^c^	0.05	0.02
Adverbs	−0.08^d^	−0.04	0.03	0.02
Adjectives	0.19^c^	0.02	−0.04	−0.03
Topic spread (averages)				
Lexical chains	0.21^c^	−0.02	−0.26^d^	−0.27^d^
Lexical chain length	0.10^d^	0.06	−0.34^c^	−0.36^c^
Lexical chain span	0.17^c^	−0.07	−0.24^c^	−0.25^c^
Lexical cross chains	0.21^c^	−0.05	−0.26^c^	−0.27^c^

a MC: multiple-choice.

b TF: true-false.

c The correlation is significant at a significance level of 0.01 (2-tailed).

d The correlation is significant at a significance level of 0.0001 (2-tailed).

Table 3 shows the results for the audio condition. Average word count shows a strong positive correlation with perceived difficulty, with correlations of 0.11 for MC and a negative correlation with information retention (−0.23 for exact word matching and −0.25 for similar word matching), indicating that shorter texts are perceived as easier. When looking at the first group of features, the content word frequency has a significant negative correlation (−0.20) with perceived difficulty (more common words are seen as easier) and higher information retention. Higher content word frequency is also associated with better information retention (0.14 and 0.13). Grammar frequency slightly lowers perceived difficulty (−0.08) but has no significant effect on information retention.

The second group of features shows that specificity, which represents the use of precise medical terms, increases perceived difficulty (0.06) and reduces information retention (−0.21 and −0.22). Ambiguity mirrors this pattern, indicating that unclear or generalized terms also hinder comprehension and retention. Concept density and topic density, which measure the amount of information packed into an audio, correlate positively with perceived difficulty (0.27) and negatively impact information retention, with values of −0.30 and −0.33 for concept density and −0.39 and −0.42 for topic density. The third group of features shows that a higher percentage of nouns increases perceived difficulty (0.20), although the effect on recall is minimal (0.07 for exact word matching). Verbs, on the other hand, decrease the perceived difficulty of the audio (−0.29), though the impact on recall is negligible (0.02). Adjectives slightly increase perceived difficulty (0.18), while adverbs reduce it (−0.13), but neither significantly affects retention. The fourth group of features shows that audio with more lexical chains (indicating distinct topics) correlates with increased perceived difficulty (0.15) and reduced information retention (−0.25 and −0.27). Lexical chain span, representing the extent of topic coverage, similarly increases perceived difficulty (0.12) and negatively impacts retention (−0.24 and −0.25). Lexical cross chains, which measure topic overlap, follow a similar trend, correlating positively with perceived difficulty (*r*=0.15) and negatively with retention, while showing a slight benefit for MC performance (*r*=0.06). Overall, the findings suggest that the impact of text features on difficulty varies somewhat between text and audio delivery. Participants perceive dense, health care–specific content as more difficult in text form, but the effects on actual difficulty and recall are mixed across both modalities.

**Table 3.** Correlation of text features with the dependent variables for the strict dataset (information presented as audio).

Features	Perceived difficulty	Actual difficulty		
		Comprehension	Information retention	
		MC^a^ and TF^b^	Percentage of exact matching words	Percentage of similar words
Average word count	0.11^c^	0.03	−0.23^c^	−0.25^c^
Ordinariness				
Content word frequency	−0.20^c^	0.01	0.14^c^	0.13^c^
Grammar frequency	−0.08^d^	0.03	−0.01	−0.01
Health care domain specialty (averages)				
Specificity	0.06^e^	0.02	−0.21^c^	−0.22^c^
Ambiguity	0.25^c^	0.08^d^	−0.30^c^	−0.32^c^
Concept density	0.27^c^	0.07^d^	−0.30^c^	−0.33^c^
Topic density	0.27^c^	0.07^d^	−0.39^c^	−0.42^c^
Parts-of-speech features (%)				
Nouns	0.20^c^	0.11^d^	0.07^e^	0.04
Verbs	−0.29^c^	−0.11^c^	0.02	0.03
Adverbs	−0.13^c^	−0.03	0.01	0.01
Adjectives	0.18^c^	0.11^c^	−0.04	−0.04
Topic spread (averages)				
Lexical chains	0.15^c^	0.06^e^	−0.25^c^	−0.27^c^
Lexical chain length	−0.03	0.01	−0.05	−0.06^e^
Lexical chain span	0.12^c^	0.03	−0.24^c^	−0.25^c^
Lexical cross chains	0.15^c^	0.06^e^	−0.25^c^	−0.27^c^

a MC: multiple-choice.

b TF: true-false.

c The correlation is significant at a significance level of 0.0001 (2-tailed).

d The correlation is significant at a significance level of 0.01 (2-tailed).

e The correlation is significant at a significance level of 0.05 (2-tailed).

### Follow-Up Analysis

#### Education Level

**Figure 5.**
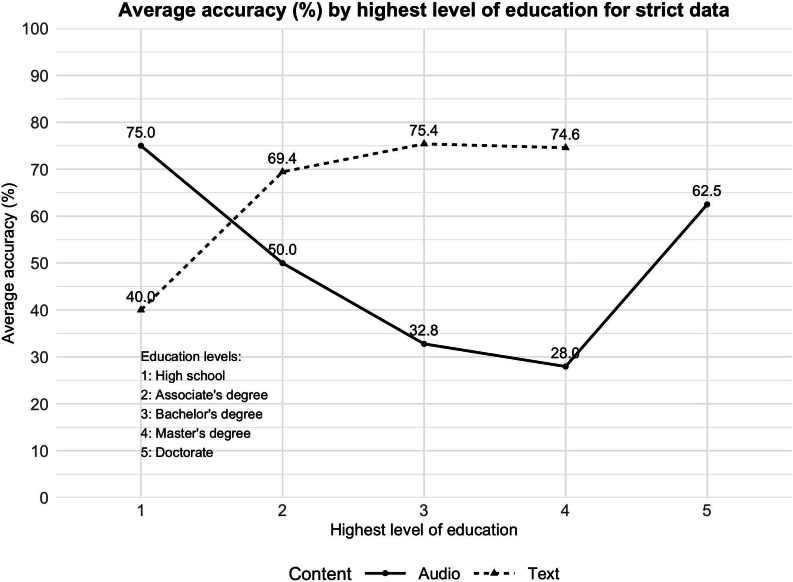
Average comprehension (%; multiple-choice and true-false) results by education level.

We further analyzed the results by participants’ education levels to assess how comprehension (measured via MC and true/false questions) varied across educational backgrounds. Figure 5 illustrates average comprehension accuracy by education level, where Level 1 represents high school to Level 5 represents doctoral education. The figure presents results separately for audio and text conditions for the strict dataset.

Overall, comprehension scores tend to peak at midlevel education (level 3 or 4) and decline slightly at the highest education level (level 5) for the text condition. In the audio condition, average accuracy is highest for participants with a high school degree (levels 2 and 3), reaching 75%. Accuracy drops noticeably at higher levels: to 32.8% for level 3 and 28% for level 4. The lenient dataset follows a similar trend with slightly higher scores (Multimedia Appendix 1).

In the text condition, accuracy is consistently high for levels 2 through 4. There were no participants with an education level 5 for the text condition. These patterns suggest that while moderate educational attainment supports better comprehension of health information, the highest education group may pay less attention or assume prior familiarity, resulting in lower scores. These findings highlight the importance of tailoring health information not just by readability level but also by considering the potential attention patterns and assumptions made by individuals with different education levels.

#### English Spoken at Home

**Figure 6.**
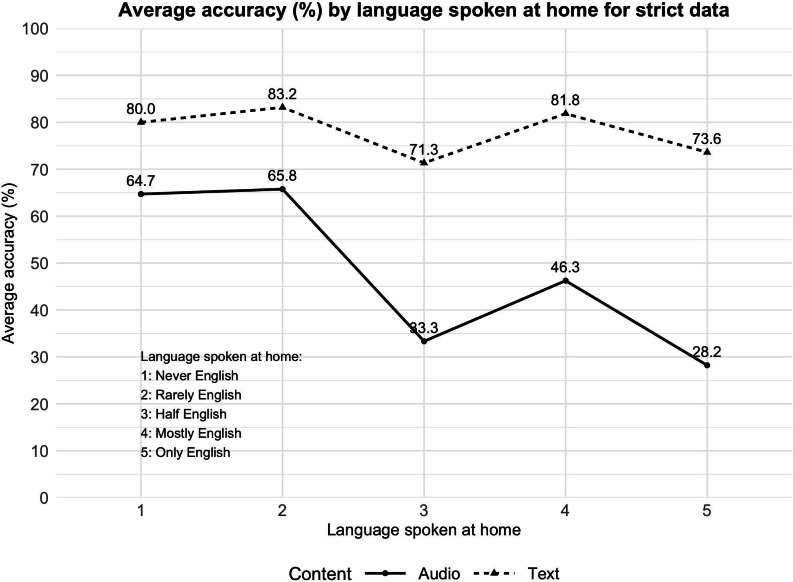
Average comprehension (%) by English spoken at home.

We also examined comprehension scores by participants’ English language use at home, as shown in Figure 6. Language levels range from level 1 (Never English at home) to level 5 (Only English at home). In the audio condition, comprehension was highest at level 2 (Rarely English), with 65.8% accuracy. However, scores dropped significantly for levels 3 through 5, with level 5 (Only English) showing the lowest average accuracy at 28.2%. This unexpected pattern may reflect that nonnative speakers were more attentive when processing audio content, compensating for language challenges by focusing more during listening.

In contrast, the text condition showed more consistent performance across language levels, with scores remaining relatively stable. The strict dataset shows the highest comprehension at level 2 (83.2%) and level 4 (81.8%), while level 5 participants (Only English at home) scored 73.6%. The lenient dataset shows similar results as well (Multimedia Appendix 1). This suggests that while nonnative speakers may show high attentiveness in both modalities, native speakers may slightly underperform due to familiarity or less cognitive engagement. These results emphasize the need to consider not only content complexity but also users’ language backgrounds and attentional behavior when designing health information for diverse audiences.

#### Readability Formulas

We have analyzed the relationship between existing readability formulas, Flesch Reading Ease, Gunning Fog Index, Smog Index, Dale-Chall Readability Score, and perceived and actual difficulty. We apply a Bonferroni correction since these were not a priori posed hypotheses. Results with *P*≤.03 are considered significant after the correction.

For the text condition (Table 4), the readability formulas are aligned with the perceived difficulty results. The Flesch Reading Ease has a significant negative correlation (−0.292), indicating that as texts become simpler, they are perceived as less difficult, while the Smog Index shows a strong positive correlation (0.334), indicating that higher difficulty increases perceived difficulty. The Gunning Fog Index (0.201) and Dale-Chall Readability Score (0.196) also positively correlate with perceived difficulty, but to a lesser degree.

For actual difficulty, the Smog Index provides significant results for information retention. There are no significant results for the correlation with comprehension. The Flesch Reading Ease shows a significant positive correlation with information retention (0.15 and 0.126). This result suggests retention is higher when content is easier to understand, according to the Flesch Reading Ease. Conversely, the Smog Index has strong negative correlations with information retention (−0.336 and −0.337). This result suggests retention is higher when content is easier to understand according to the Smog Index. For the audio condition (Table 5), we see only significant correlations for the Smog Index and free recall. It has strong negative correlations with free recall (−0.12 and −0.14).

**Table 4.** Correlation of readability metrics with the dependent variables for the strict dataset: information presented as text.

Readability	Perceived difficulty	Actual difficulty		
		Comprehension	Information retention	
		MC^a^ and TF^b^	Percentage of matching words	Percentage of similar words
Flesch reading ease	−0.29^c^	−0.03	0.15^c^	0.13^c^
Gunning Fog Index	0.20^c^	0.05	−0.05	−0.03
Smog Index	0.33^c^	0.01	−0.34^c^	−0.34
Dale-Chall readability score	0.20^c^	0.02	0.016	0.05

a MC: multiple-choice.

b TF: true-false.

c The correlation is significant at a significance level of 0.0001 (2-tailed).

**Table 5.** Correlation of readability metrics with the dependent variables for the strict dataset: information presented as audio.

Readability	Perceived difficulty	Actual difficulty		
		Comprehension	Information retention	
		MC^a^ and TF^b^	Percentage of matching words	Percentage of similar words
Flesch reading ease	0.08	0.07	0.07	0.07
Gunning Fog Index	−0.09	−0.07	−0.06	−0.05
Smog Index	0.04	−0.05	−0.12^c^	−0.14^d^
Dale-Chall readability score	−0.07	−0.05	−0.01	0.00

a MC: multiple-choice.

b TF: true-false.

c The correlation is significant at a significance level of 0.001 (2-tailed).

d The correlation is significant at a significance level of 0.0001 (2-tailed).

## Discussion

### Principal Findings

Our study provides comprehensive insights into how text and audio modalities influence health information comprehension and retention. Consistent with prior research on health literacy, our results indicate that text-based materials are more effective than audio for conveying difficult health information [34]. Text consistently yielded higher comprehension and retention scores, particularly for content with elevated domain-specific complexity, such as the *BMJ* journal articles. These findings align with established literature suggesting that individuals process written health information more thoroughly, possibly due to its static nature and the ability to reread content at their own pace [20,34,35].

The strength of text-based communication was further emphasized through information retention scores. Exact word matching and semantic similarity measures demonstrated significantly better retention with text. For example, *BMJ* journal texts resulted in nearly triple the scores compared with their audio counterparts. These results support the idea that reading facilitates both literal and conceptual memory performance, an essential aspect of health literacy that encompasses access to information and the ability to process and act on it.

In contrast, audio demonstrated potential benefits for easier content. Sources like WebMD and Patient Instructions showed more comparable performance between modalities, with audio occasionally yielding slightly better perceived difficulty scores. This finding aligns with the growing body of work suggesting that audio can effectively deliver conversational or familiar health content, particularly when designed to match natural speech patterns [28]. It also reflects the rise of voice-assisted health tools and smart speakers in disseminating accessible health information.

One notable contribution of this study is the documented divergence between perceived and actual difficulty. Although participants often rated text as easier to understand, these ratings were not always associated with actual comprehension. For instance, Simple Wikipedia texts were perceived as easy across both modalities, but their actual comprehension and retention varied.

Furthermore, the text feature-level analysis revealed how specific linguistic features affect difficulty across modalities. Higher content word frequency was associated with lower perceived difficulty and better retention in both text and audio. Verb-rich content improved comprehension, especially in audio, likely due to its alignment with speech patterns. In contrast, noun and adjective-heavy texts were more challenging to process, particularly in audio. These findings support prior work suggesting that lexical familiarity and syntactic simplicity enhance understanding in health communication [25].

Health care–specific features such as concept density, ambiguity, and topic density were positively correlated with perceived difficulty and negatively with retention across both modalities. These metrics reflect the cognitive burden of specialized vocabulary and dense informational content. Results support earlier findings that medical information becomes more difficult when overloaded with abstract or specialized terms. Our results show that these challenges are exacerbated in audio formats, where listeners cannot visually parse or revisit complex phrases.

The evaluation of traditional readability formulas revealed that while these tools moderately correlate with perceived difficulty in text, they are ineffective in determining actual difficulty. The Smog Index showed a notable correlation with information retention, but overall, readability scores failed to account for comprehension or retention outcomes in audio. This highlights a significant gap in current health communication tools, which are often developed with text readability in mind and overlook the unique cognitive demands of audio-based information processing.

Follow-up analyses on demographics added further nuance to our findings. Participants with moderate levels of education outperformed those with the highest levels in both modalities. One plausible explanation is that more highly educated individuals may skim or assume familiarity with the content, resulting in lower attention and performance. Similarly, participants who rarely used English at home performed well in the audio condition, possibly due to heightened attention or greater effort in processing nonnative spoken information. These patterns suggest that educational background and language exposure play essential roles in how health information is consumed and understood, a point echoed in health literacy studies focusing on underserved and multilingual populations.

In addition, the corpora used in this study had unequal sample sizes (eg, WebMD and Patient Instructions [n=40] vs Simple Wikipedia [n=243]), primarily due to technical and access constraints. However, all text feature calculations were performed at the individual document level, and our comparative analysis focused on linguistic and syntactical differences among corpora rather than pooled averages. To assess the potential influence of this imbalance, we examined all documents for extreme feature values that might bias results and found none with extreme values.

### Limitations

Our findings offer initial evidence to suggest that presenting complex health information in text format contributes to greater audience comprehension compared with audio format. However, it also reveals nuances, suggesting that optimized health information communication requires more than just improving readability: it demands careful alignment with content complexity, delivery modality, and consideration of audience characteristics.

While our findings offer valuable insights, several limitations must be acknowledged. We assume that Mechanical Turk workers possess a baseline level of digital literacy, as participation requires the use of internet-connected devices, familiarity with the AMT platform, and the ability to navigate and complete web-based tasks independently. Although AMT enables access to a diverse and geographically dispersed participant pool, it does not control participant selection. Participants self-select the tasks they wish to complete. As a result, our sample population was not fully representative of the general population. Specifically, more participants in our study were male (approximately 67%) and most were White (approximately 95%). In addition, constraining the participants to individuals with comparable characteristics limits extensive interparticipant variances that may attenuate the ability to identify differences in presentation format and text characteristics. We are particularly sensitive to the possibility that some of the source texts, both from technical journals and more lay-focused items, may not be culturally sensitive to many population subgroups because they were not captured by the text characterization parameters shown in Table 1.

### Future Directions

Future studies should investigate how factors such as health literacy and learning preferences influence comprehension across modalities. Integrating images with text and audio remains an important direction for our ongoing research agenda. Moreover, recruiting more demographically representative and clinically relevant populations, such as patients with direct health concerns and older adults, could offer deeper insights and lead to more personalized and effective health communication strategies.

### Conclusions

Our findings underscore the importance of tailoring health information delivery to content difficulty and user characteristics such as modality preference, vocabulary familiarity, and linguistic background. The text offers significant advantages for comprehension and retention, especially when the content is difficult or technical. Audio can complement text, particularly for familiar or conversational content, but should be carefully designed, especially for nonnative speakers or individuals with limited health literacy. Our study contributes to a more nuanced understanding of health literacy across modalities by integrating corpus-level analysis, participant outcomes, and content features. This highlights the need for modality-specific design principles and evaluation metrics to support informed decision-making, improve patient outcomes, and foster equitable access to health information in increasingly digital health care environments.

## Supplementary material

10.2196/69772 Multimedia Appendix 1 Additional tables and text samples.

## Abbreviations

AMT: Amazon Mechanical Turk
*BMJ*: *British Medical Journal*
HIT: human intelligence task
*ICD-10*: *International Statistical Classification of Diseases, Tenth Revision*
IRB: Institutional Review Board
MC: multiple-choice
TF: true-false

## References

[1] Eichler K, Wieser S, Brügger U (2009). The costs of limited health literacy: a systematic review. Int J Public Health.

[2] Hart TL, Blacker S, Panjwani A, Torbit L, Evans M (2015). Development of multimedia informational tools for breast cancer patients with low levels of health literacy. Patient Educ Couns.

[3] Koh HK, Berwick DM, Clancy CM (2012). New federal policy initiatives to boost health literacy can help the nation move beyond the cycle of costly ‘Crisis Care’. Health Aff (Millwood).

[4] (2019). Plain language summit 2019: a day of talks and conversations on how to advance plain language in government communications. Digital.gov.

[5] Grene M, Cleary Y, Marcus-Quinn A (2017). Use of plain-language guidelines to promote health literacy. IEEE Trans Profess Commun.

[6] Choi S, Nanda P, Yuen K, Ong K (2023). Bridging the gap in health literacy research: the inclusion of individuals with visual impairments. Patient Educ Couns.

[7] Thormundsson B (2023). Number of voice assistant users in the United States from 2022 to 2026. Statista.

[8] Leibler S (2019). Cedars-sinai taps alexa for smart hospital room pilot. Cedars-Sinai.

[9] Yoo TK, Oh E, Kim HK (2020). Deep learning-based smart speaker to confirm surgical sites for cataract surgeries: a pilot study. PLoS One.

[10] (2022). Voice tech in healthcare: transformation and growth. Modev.

[11] Sun W, Min X, Lu W, Zhai G A deep learning based no-reference quality assessment model for ugc videos.

[12] DuBay W (2004). The Principles of Readability.

[13] Berkman ND, Sheridan SL, Donahue KE, Halpern DJ, Crotty K (2011). Low health literacy and health outcomes: an updated systematic review. Ann Intern Med.

[14] Mackert M, Mabry-Flynn A, Champlin S, Donovan EE, Pounders K (2016). Health literacy and health information technology adoption: the potential for a new digital divide. J Med Internet Res.

[15] Nutbeam D (2008). The evolving concept of health literacy. Soc Sci Med.

[16] Rudd RE (2015). The Evolving Concept of Health Literacy: New Directions for Health Literacy Studies.

[17] Mayer GG, Villaire MM (2007). Health Literacy in Primary Care: A Clinician’s Guide.

[18] Badarudeen S, Sabharwal S (2010). Assessing readability of patient education materials: current role in orthopaedics. Clin Orthop Relat Res.

[19] (2021). Flesch reading ease and the flesch kincaid grade level. Readable.

[20] Leroy G, Helmreich S, Cowie JR, Miller T, Zheng W (2008). Evaluating online health information: beyond readability formulas. AMIA Annu Symp Proc.

[21] Brants T, Franz A (2006). The google web 1T 5-gram corpus version 1.1. Http://www.ldc.upenn.edu/Catalog/CatalogEntry.jsp?catalogId=LDC2006T13.

[22] Kauchak D, Leroy G, Hogue A (2017). Measuring text difficulty using parse-tree frequency. J Assoc Inf Sci Technol.

[23] Kauchak D, Mouradi O, Pentoney C, Leroy G Text simplification tools: using machine learning to discover features that identify difficult text.

[24] Mukherjee P, Leroy G, Kauchak D (2019). Using lexical chains to identify text difficulty: a corpus statistics and classification study. IEEE J Biomed Health Inform.

[25] Leroy G, Kauchak D, Harber P, Pal A, Shukla A (2024). Text and audio simplification: human vs. ChatGPT. AMIA Jt Summits Transl Sci Proc.

[26] McNamara DS (2014). Automated Evaluation of Text and Discourse with Coh-Metrix.

[27] Ahmed A, Leroy G, Lee S Influence of audio speech rate and source text difficulty on health information comprehension and retention.

[28] Ahmed A, Leroy G, Rains SA, Harber P, Kauchak D, Barai P (2024). Effects of added emphasis and pause in audio delivery of health information. AMIA Jt Summits Transl Sci Proc.

[29] Kang O, Thomson RI, Moran M (2018). Empirical approaches to measuring the intelligibility of different varieties of english in predicting listener comprehension. Lang Learn.

[30] Sulem E, Abend O, Rappoport A (2018). Semantic structural evaluation for text simplification. arXiv.

[31] Brämer GR (1988). International statistical classification of diseases and related health problems. tenth revision. World Health Stat Q.

[32] Leroy G, Endicott JE, Kauchak D, Mouradi O, Just M (2013). User evaluation of the effects of a text simplification algorithm using term familiarity on perception, understanding, learning, and information retention. J Med Internet Res.

[33] Leroy G, Endicott JE Combining NLP with evidence-based methods to find text metrics related to perceived and actual text difficulty.

[34] Leroy G, Kauchak D, Haeger D, Spegman D (2022). Evaluation of an online text simplification editor using manual and automated metrics for perceived and actual text difficulty. JAMIA Open.

[35] Mikolov T (2013). Efficient estimation of word representations in vector space. arXiv.

[36] Barai P, Leroy G, Bisht P (2024). Crowdsourcing with enhanced data quality assurance: an efficient approach to mitigate resource scarcity challenges in training large language models for healthcare. AMIA Jt Summits Transl Sci Proc.

