# Supplementary material for: Parallel Corpus Analysis of Text and Audio Comprehension to Evaluate Readability Formula Effectiveness: Quantitative Analysis
Source: J Med Internet Res. 2025 Oct 2;27:e69772. doi: 10.2196/69772 (PMC12490814; doi:10.2196/69772)
Supplement: Multimedia Appendix 1 [file jmir-v27-e69772-s001.docx]

**Table S1.** Demographic information (Lenient Dataset)

|  | **Lenient** | |
| --- | --- | --- |
|  | **Audio** | **Text** |
| **Characteristic** | N (%) | N (%) |
| **Count** | 239 | 269 |
| **Sex** |  |  |
| Male | 161 (67.36) | 178 (66.17) |
| Female | 76 (31.79) | 91 (33.82) |
| Other | 0 (0) | 0 (0) |
| **Age** |  |  |
| Younger than 30 years old | 76 (31.79) | 80 (29.73) |
| 30 to 39 years old | 118 (49.37) | 142 (52.78) |
| 40 to 49 years old | 22 (9.20) | 26 (9.66) |
| 50 to 59 years old | 16 (6.69) | 14 (5.20) |
| 60 to 69 years old | 6 (2.51) | 6 (2.23) |
| 70 to 79 years old | 1 (0.41) | 1 (0.37) |
| **Race** |  |  |
| Asian | 8 (3.34) | 3 (1.11) |
| American Indian/ Native Alaskan | 1 (0.41) | 1 (0.37) |
| Black or African American | 1 (0.41) | 2 (0.7) |
| Native Hawaiian or other Pacific Islander | 0 (0) | 0 (0) |
| White | 225 (91.14) | 259 (96.28) |
| Asian & White | 3 (1.25) | 4 (1.48) |
| **Ethnicity** |  |  |
| Hispanic or Latino | 76 (31.79) | 64 (23.79) |
| Not Hispanic or Latino | 162 (67.78) | 205 (76.20) |
| **Education** |  |  |
| Less Than High School | 0 (0) | 0 (0) |
| High School | 2 (0.83) | 3 (1.11) |
| Associate's degree | 2 (0.83) | 1 (0.37) |
| Bachelor's degree | 180 (75.31) | 216 (80.29) |
| Master's Degree | 52 (21.75) | 48 (17.84) |
| Doctorate Degree | 0 (0) | 0 (0) |
| Other Professional Degree | 0 (0) | 0 (0) |
| **English Speaking** |  |  |
| Never English | 7 (2.92) | 2 (0.74) |
| Rarely English | 7 (2.92) | 6 (2.23) |
| Half English | 6 (2.51) | 8 (2.97) |
| Mostly English | 27 (11.29) | 32 (11.89) |
| Only English | 191 (79.91) | 221 (82.15) |

**Table S2:** Demographic information (Strict Dataset)

|  | | Strict Dataset | |
| --- | --- | --- | --- |
|  | | Audio | Text |
| Characteristic | | N (%) | N (%) |
| Total | | 213 | 274 |
| **Sex** | |  |  |
|  | Male | 143 (67.13) | 185 (67.51) |
|  | Female | 69 (32.39) | 89 (32.48) |
|  | Other | 0 (0) | 0 (0) |
| **Age** | |  |  |
|  | 18 to 29 years old | 71 (33.33) | 79 (28.83) |
|  | 30 to 39 years old | 103 (48.35) | 151 (55.10) |
|  | 40 to 49 years old | 19 (8.92) | 25 (9.12) |
|  | 50 to 59 years old | 13 (6.10) | 12 (4.37) |
|  | 60 to 69 years old | 6 (2.81) | 6 (2.18) |
|  | 70 to 79 years old | 1 (0.46) | 1 (0.36) |
| **Race** | |  |  |
|  | Asian | 6 (2.81) | 3 (1.09) |
|  | American Indian/ Native Alaskan | 1 (0.46) | 1 (0.36) |
|  | Black or African American | 1 (0.46) | 2 (0.72) |
|  | Native Hawaiian or other Pacific Islander | 0 (0) | 0 (0) |
|  | White | 199 (94.83) | 263 (95.98) |
|  | Asian & White | 6 (2.81) | 5 (1.82) |
| **Ethnicity** | |  |  |
|  | Hispanic or Latino | 60 (28.16) | 72 (26.27) |
|  | Not Hispanic or Latino | 153 (71.83) | 202 (73.72) |
| **Education (Highest Degree Achieved)** | |  |  |
|  | Less Than High School | 0 (0) | 0 (0) |
|  | High School | 2 (0.93) | 2 (0.72) |
|  | Associate's degree | 2 (0.93) | 1 (0.36) |
|  | Bachelor's degree | 165 (77.46) | 214 (78.10) |
|  | Master's Degree | 43 (20.18) | 56 (20.43) |
|  | Doctorate Degree | 0 (0) | 0 (0) |
|  | Other Professional Degree | 0 (0) | 0 (0) |
| **English Speaking** | |  |  |
|  | Never English at Home | 6 (2.81) | 2 (0.72) |
|  | Rarely English at Home | 7 (3.28) | 6 (2.18) |
|  | Half of the time English at Home | 6 (2.81) | 7 (2.55) |
|  | Mostly English at Home | 21 (9.85) | 41 (14.96) |
|  | Only English at Home | 173 (81.22) | 218 (79.56) |

**Table S3.** Perceived Difficulty for lenient dataset. (A lower value means perceived as easier)

|  | **Perceived Difficulty** | |
| --- | --- | --- |
| **Test Sources (N)** | **Audio (SD)** | **Text (SD)** |
| **BMJ Lay Summary (193 texts) ****** | 2.08 (1.1) | 1.83 (1.1) |
| **WebMD (40 texts)** | 1.97 (0.9) | 2.08 (1.0) |
| **Patient instruction (40 texts)** | 2 (1.0) | 1.79 (1.2) |
| **Simple Wikipedia (243 texts)** | 2.16 (1.1) | 2.14 (1.1) |
| **BMJ Journal (200 texts) *** | 2.82 (1.1) | 2.52 (1.0) |
| **Overall *** | 2.30 (1.0) | 2.14 (1.1) |

(Significance, * = p < 0.05, ** = p < 0.01, *** = p < 0.001, **** = p < 0.0001)

**Table S4.** Accuracy (%) for MC and TF questions for lenient dataset. (A higher value means better comprehension)

|  | **Actual Difficulty** | |
| --- | --- | --- |
| **Test Sources (N)** | **Audio (SD)** | **Text (SD)** |
| **BMJ Lay Summary (193 texts) ****** | 55.49 (37) | 67.42 (34) |
| **WebMD (40 texts) ****** | 53.51 (38) | 75.53 (32) |
| **Patient instruction (40 texts) ***** | 58.59 (32) | 71.5 (29) |
| **Simple Wikipedia (243 texts) ****** | 64.61 (39) | 73.8 (36) |
| **BMJ Journal (200 texts) ***** | 57.81 (36) | 72.14 (33) |
| **Overall ***** | 59.30 (37) | 71.58 (34) |

(Significance, * = p < 0.05, ** = p < 0.01, *** = p < 0.001, **** = p < 0.0001)

**Table S5.** Exact matching word (%) of information retention for lenient dataset. (A higher value means better recall)

|  | **Number of exact words recalled (Lenient Dataset)** | |
| --- | --- | --- |
| **Test Sources (N)** | **Audio (SD)** | **Text (SD)** |
| **BMJ Lay Summary (193 texts)** | 8.9 (5) | 10.06 (6) |
| **WebMD (40 texts)** | 7.61 (5) | 6.6 (5) |
| **Patient instruction (40 texts)** | 4.21 (8) | 6.12 (6) |
| **Simple Wikipedia (243 texts) **** | 8.25 (3) | 10.84 (4) |
| **BMJ Journal (200 texts) ***** | 2.64 (7) | 6.88 (5) |
| **Overall *** | 6.60 (5) | 9.02 (5) |

(Significance, * = p < 0.05, ** = p < 0.01, *** = p < 0.001, **** = p < 0.0001)

**Table S6.** Similar word (%) of Free Recall for lenient dataset. (A higher value means better recall)

|  | **Number of similar words recalled (Lenient Dataset)** | |
| --- | --- | --- |
| **Test Sources (N)** | **Audio (SD)** | **Text (SD)** |
| **BMJ Lay Summary (193 texts)** | 10.64 (6) | 11.54 (6) |
| **WebMD (40 texts)** | 9.25 (6) | 7.83 (5) |
| **Patient instruction (40 texts)** | 5.89 (10) | 6.57 (7) |
| **Simple Wikipedia (243 texts) **** | 9.58 (5) | 11.91(6) |
| **BMJ Journal (200 texts) ***** | 3.46 (4) | 7.85 (4) |
| **Overall *** | 7.93 (5) | 10.15 (5) |

(Significance, * = p < 0.05, ** = p < 0.01, *** = p < 0.001, **** = p < 0.0001)

**Table S7.** Correlation of text features with the dependent variables for lenient dataset (Audio study)

| **Features** | **Perceived Difficulty** | **Actual Difficulty** | | |
| --- | --- | --- | --- | --- |
|  |  | **Comprehension** | **Information Retention** | |
|  |  | **MC and TF** | **Percentage of Exact Matching Words** | **Percentage of Similar Words** |
| **Average Word Count** | 0.11**** | 0.03 | -0.23**** | -0.25**** |
| **Ordinariness** | | |  |  |
| **Content Word Frequency** | -0.20**** | 0.01 | 0.14**** | 0.13**** |
| **Grammar Frequency** | -0.08** | 0.03 | -0.01 | -0.01 |
| **Healthcare Domain Specialty (Averages)** | | |  |  |
| **Specificity** | 0.06* | 0.02 | -0.21**** | -0.22**** |
| **Ambiguity** | 0.25**** | 0.08** | -0.30**** | -0.32 **** |
| **Concept Density** | 0.27**** | 0.07** | -0.30**** | -0.33**** |
| **Topic Density** | 0.27**** | 0.07** | -0.39**** | -0.42**** |
| **Parts-Of-Speech Features (%)** | | |  |  |
| **Nouns** | 0.20**** | 0.11*** | 0.07* | 0.04 |
| **Verbs** | -0.29**** | -0.11**** | 0.02 | 0.03 |
|  |  |  |  |  |
| **Adverbs** | -0.13**** | -0.03 | 0.01 | 0.01 |
| **Adjectives** | 0.18**** | 0.11**** | -0.04 | -0.04 |
| **Topic Spread (Averages)** | | |  |  |
| **Lexical Chains** | 0.15**** | 0.06* | -0.25**** | -0.27**** |
| **Lexical Chain Length** | -0.03 | 0.01 | -0.05 | -0.06* |
| **Lexical Chain Span** | 0.12**** | 0.03 | -0.24**** | -0.25**** |
| **Lexical Cross Chains** | 0.15**** | 0.06* | -0.25**** | -0.27**** |

(Significance, * = p < 0.05, ** = p < 0.01, *** = p < 0.001, **** = p < 0.0001)

**Table S8.** Correlation of text features with the dependent variables for lenient dataset (Text study)

| **Features** | **Perceived Difficulty** | **Actual Difficulty** | | |
| --- | --- | --- | --- | --- |
|  |  | **Comprehension** | **Information Retention** | |
|  |  | **MC and TF** | **Percentage of Exact Matching Words** | **Percentage of Similar Words** |
|  | **Lenient** | **Lenient** | **Lenient** | **Lenient** |
| **Average Word Count** | 0.10**** | 0.01 | -0.24**** | -0.25**** |
| **Ordinariness** | | |  |  |
| **Content Word Frequency** | -0.28**** | 0.11**** | 0.09*** | 0.09*** |
| **Grammar Frequency** | -0.10**** | -0.021 | -0.01 | -0.01 |
| **Healthcare Domain Specialty (Averages)** | | |  |  |
| **Specificity** | 0.05* | 0.02 | -0.22**** | -0.23**** |
| **Ambiguity** | 0.23**** | -0.01 | -0.28**** | -0.31**** |
| **Concept Density** | 0.25**** | -0.01 | -0.30**** | -0.32**** |
| **Topic Density** | 0.26**** | -0.02 | -0.39**** | -0.42 **** |
| **Parts-Of-Speech Features (%)** | | |  |  |
| **Nouns** | 0.19**** | 0.04 | 0.08** | 0.05* |
| **Verbs** | -0.28**** | -0.03 | -0.02 | -0.01 |
|  |  |  |  |  |
| **Adverbs** | -0.09**** | 0.05 | -0.06**** | -0.06**** |
| **Adjectives** | 0.16**** | 0.02 | -0.02 | -0.02 |
| **Topic Spread (Averages)** | | |  |  |
| **Lexical Chains** | 0.14**** | 0.02 | -0.25**** | -0.27**** |
| **Lexical Chain Length** | -0.03 | -0.02 | -0.06* | -0.07** |
| **Lexical Chain Span** | 0.10**** | 0.03 | -0.24**** | -0.25**** |
| **Lexical Cross Chains** | 0.14**** | 0.02 | -0.25**** | -0.27**** |

(Significance, * = p < 0.05, ** = p < 0.01, *** = p < 0.001, **** = p < 0.0001)

**Figure S1.** Average comprehension (MC and TF) results by education level. A higher value means better result.


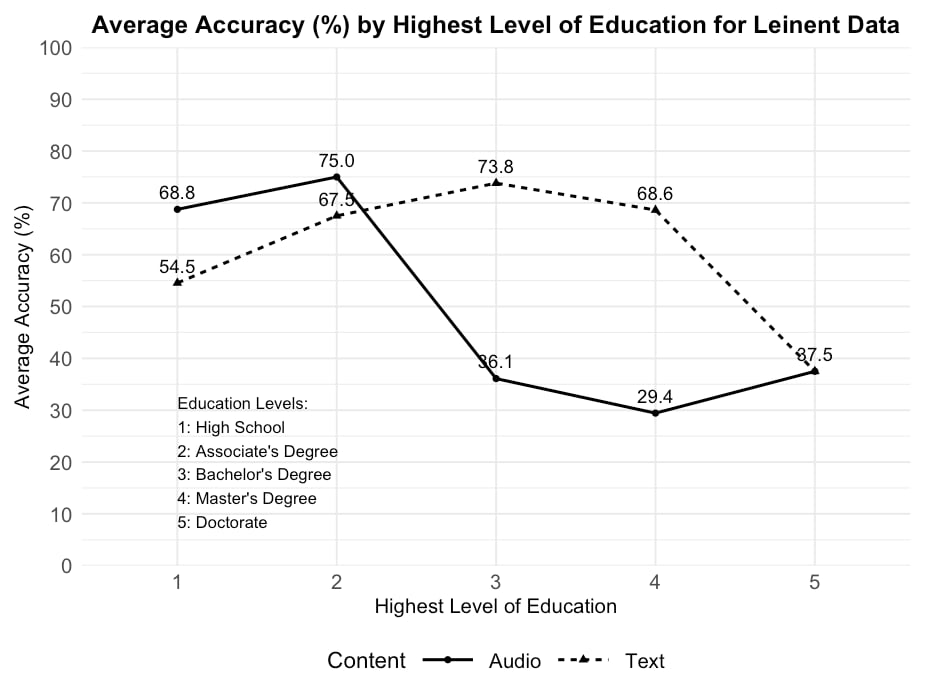


**Figure S2.** Average comprehension (%) by English spoken at home for lenient dataset. A higher value means better result.


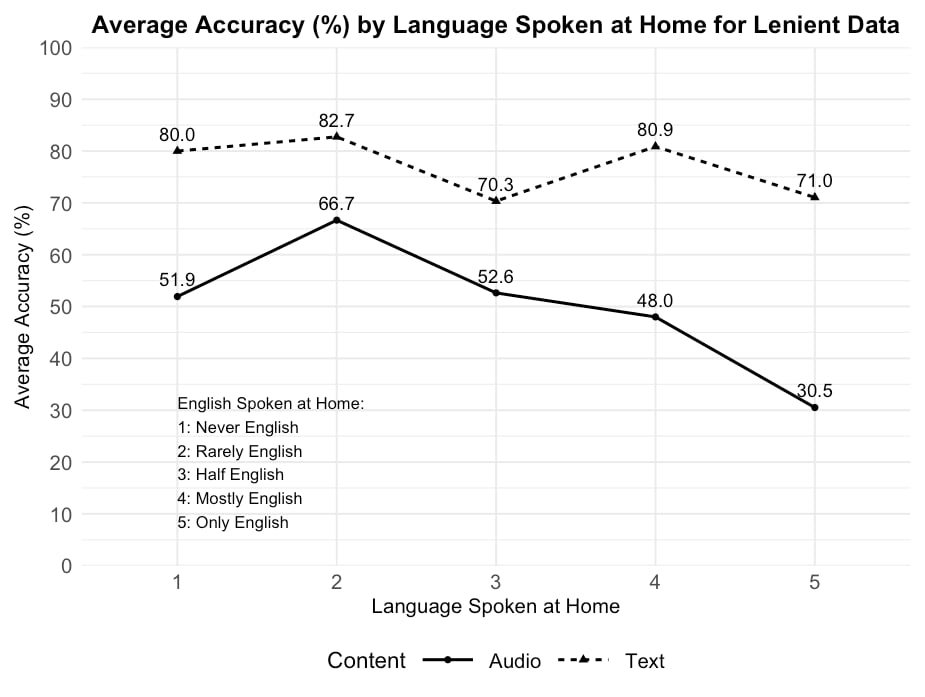


**Table S9.** Correlation of readability metrics with the dependent variables for lenient dataset information presented as text

| **Readability** | **Perceived Difficulty** | **Actual Difficulty** | | |
| --- | --- | --- | --- | --- |
|  |  | **Comprehension** | **Information Retention** | |
|  |  | **MC and TF** | **Percentage of Exact Matching Words** | **Percentage of Similar Words** |
| **Flesch Reading Ease** | -0.21 **** | -0.04 | 0.05 | 0.03 |
| **Gunning Fog Index** | 0.15 **** | 0.03 | 0.01 | 0.04 |
| **Smog Index** | 0.25 **** | 0.22 **** | -0.22 **** | -0.23 **** |
| **Dale-Chall Readability Score** | 0.16 **** | -0.10 **** | 0.09 *** | 0.12 **** |

(Significance, * = p < 0.05, ** = p < 0.01, *** = p < 0.001, **** = p < 0.0001)

**Table S10.** Correlation of readability metrics with the dependent variables for lenient dataset: information presented as audio

| **Readability** | **Perceived Difficulty** | **Actual Difficulty** | | |
| --- | --- | --- | --- | --- |
|  |  | **Comprehension** | **Information Retention** | |
|  |  | **MC and TF** | **Percentage of Exact Matching Words** | **Percentage of Similar Words** |
| **Flesch Reading Ease** | -0.01 | -0.02 | 0.06 ** | 0.06 |
| **Gunning Fog Index** | -0.04 | -0.02 | -0.03 | -0.02 |
| **Smog Index** | 0.21 **** | 0.15 **** | -0.13 **** | -0.16 **** |
| **Dale Chall Readability Score** | 0.03 | 0.03 | 0.02 | 0.03 |

(Significance, * = p < 0.05, ** = p < 0.01, *** = p < 0.001, **** = p < 0.0001)

**Text S1. Representative example of text for each source**

**BMJ journal text sample 1**

Classification criteria for systemic lupus erythematosus (SLE) have been developed to ensure the inclusion of homogeneous groups of patients in clinical studies. 1 Nonetheless, these criteria are often used in clinical practice to aid diagnosis. In this regard, the Systemic Lupus International Collaborating Clinics (SLICC) 2012 criteria2 were reported to have increased sensitivity3,5 and capture more patients at the population level,6 7 as compared with the American College of Rheumatology (ACR) 1997 criteria. 8 Still, clinical diagnosis may precede classification,9 10 suggesting that especially at early stages, not all individuals with SLE will fulfil the criteria. Moreover, organ-dominant forms may occur imposing further classification challenges. Recently, the European League Against Rheumatism (EULAR) jointly with the ACR have introduced new classification criteria,11 12 which are based on two novel concepts, namely antinuclear antibodies(ANA) as an entry criterion coupled with variably weighed features. 13 Whether the new criteria have higher accuracy and allow for earlier classification merits investigation across different cohorts. The prognostic implications of classifying or not patients with SLE with the existing criteria is also not known. We compared the three classification criteria in a large cohort of patients with early diagnosis of SLE or other rheumatological diseases, spanning from the community to tertiary care. Sensitivity was determined at the time of diagnosis and also, at last follow-up; we also examined which criteria enabled earlier classification. Guided by our observation that criteria classify non- overlapping patient groups, we compared the phenotypic characteristics and outcomes among patients who are unclassified by each criterion. Based on our sample characteristics, we propose modifications in the classification algorithms to assure the highest combination of sensitivity and specificity, thus allowing earlier classification and treatment of patients with potentially high disease burden.

**BMJ journal text sample 2**

The severe lung and systemic inflammatory manifestations observed in severe acute respiratory distress syndrome coronavirus 2 (SARS-CoV-2) infection have led to the hypothesis of a hyperinflammatory mechanism, more dependent on the host response than on direct viral cellular damage. 1 2 Certain parallelisms with other cytokine storm situations, such as macrophage activation syndrome or chimeric antigen receptor T-cell-associated systemic inflammatory syndromes have been invoked in support. This hypothesis has prompted the rapid introduction of anti-inflammatory and immunomodulatory agents approved for rheumatic diseases in the therapeutic strategies to combat SARS-CoV-2 infection. The prevalence of severe SARS-CoV-2 infection in patients with previous autoimmune or inflammatory diseases is unknown. This group of patients is not represented in the largest Chinese series as a specific risk factor for susceptibility or severity of COVID-19. 3 4 An excess of morbimortality associated with the previous use of conventional or targeted immunosuppressive drugs has neither been reported. However, in an Italian series of 1591 patients with severe COVID-19 admitted to the ICU, the most prevalent comorbidity in patients under 40 years old was a miscellanea of patients that included inflammatory and immunosuppressed patients. 5 A potential preventive or therapeutic effect of certain immunomodulatory therapies in these patients has been hypothesised. Among them, antimalarials, colchicine, corticosteroids, jakinibs and interleukin (IL)-6 or IL-1 antagonists are being used under special conditions or clinical trials with weak evidence. 6 However, the risks of these drugs in the context of viral infections without concomitant antiviral therapies are not negligible. 7,9 Whether immunosuppressants put patients with rheumatic disease at an increased or decreased risk for severe COVID-19 is unknown, and evidence is urgently needed to guide prevention and therapy. 10 11 Since timely obtaining methodologically rigorous data on the prevalence of severe SARS-CoV-2 infection in our patients under different therapies is challenging at this moment,8 we have performed an exploratory analysis of the relative prevalence of hospital-diagnosed COVID-19 in large multicentric cohorts of rheumatic patients under follow-up.

**BMJ journal text sample 3**

Antineutrophil cytoplasmic antibody (ANCA)-associated vasculitis (AAV) is a small-to-medium vessel vasculitis characterised by disease relapses, increased risk of end-stage renal disease (ESRD) and excess mortality. 1 2 Most patients with AAV have circulating ANCA that target proteinase 3 (PR3) or myeloperoxidase (MPO) and are considered pathogenic. 3 ANCA testing has been a central component of AAV diagnosis since the 1980s,4 5 but the measurement of ANCA titres after treatment has been a controversial practice. Using contemporary induction strategies, the majority of patients with AAV achieve clinical remission. 6 However, only a proportion achieve concurrent serological remission with negative serum ANCA assay. 7,10 Research on the clinical utility of post-treatment ANCA measurements has generated conflicting findings, perhaps due to heterogeneous methods that have investigated variable patient groups. Some studies focused on patients with persistently positive titres, while others investigated those with rising titres or re-emerging ANCA after negative testing. 7,16 Interest in using ANCA as a biomarker for disease activity stems from its potentially pathogenic role in AAV disease and early studies suggesting that rising ANCA titre may predict disease flare and relapse. 17 18 However, a subsequent meta-analysis found that repeat ANCA testing to identify patients with rising or persistent ANCA titres had limited utility for guiding patient management. 14 Despite those findings, there was a resurgence of enthusiasm for repeat ANCA testing after the adoption of rituximab for AAV induction treatment since rituximab depletes circulating precursors to ANCA-producing immune cells and significantly decreases ANCA titres. 6 19 However, recent research, including observational studies and the Maintenance of Remission using Rituximab in Systemic ANCA-associated Vasculitis (MAINRITSAN)2 randomised clinical trial have suggested that rising ANCA titres may be specific but imperfect predictors of AAV relapse. 20 In light of these conflicting data, the impact of achieving a serological remission on later risk of relapse, ESRD and death remains unknown. To investigate the association of postinduction ANCA titers with key AAV outcomes, we emulated a target trial using observational data to examine the effect of achieving a serological remission after treatment on the subsequent risks of relapse, ESRD and death within 5 years.

**BMJ lay summary text sample 1**

Tumour necrosis factor (TNF) inhibitors, also known as biologics, are still fairly recent additions to the treatment options for RA. In the UK doctors generally only offer TNF inhibitors to people who dont do well on the more established disease-modifying anti-rheumatic drugs (DMARDs) such as methotrexate. However, their use is becoming more common and researchers are finding out more about them.

**BMJ lay summary text sample 2**

EULAR recommendations give advice to doctors, nurses and patients about the best way to treat and manage diseases. EULAR has updated its recommendations on looking after people with fibromyalgia, a rheumatic condition that is associated with widespread pain and fatigue. People with Fibromyalgia may also report headaches, migraines, non-refreshing sleep, mood disturbances and bowel or stomach problems. A group of 18 experts worked together to develop these recommendations.

**BMJ lay summary text sample 3**

Psoriatic arthritis is a chronic inflammatory disease that affects a person's joints, causing pain and disability. The disease often causes swelling of the fingers and toes. It gets its name from the link between this type of arthritis and a skin condition called psoriasis, which causes redness and scaling. Being able to measure disease activity in psoriatic arthritis allows doctors to see how well medicines are working. There are many different disease activity scores available that look at different parameters. The Disease Activity Index for Psoriatic Arthritis (also known as DAPSA) is one of these and has been developed to include a series of key disease elements. High disease activity generally means that a person's disease is not well controlled, and may be causing joint damage and pain. Being in low disease activity or remission (no signs of symptoms) is the goal of treatment for psoriatic arthritis.

**Patient instruction text sample 1**

These instructions are for Children's Hospital of Philadelphia (CHOP) patients
with a peripheral intravenous catheter (PIV). Important information:

A PIV is inserted when your child needs intravenous (IV) fluids or IV medicines that cannot be given by mouth or feeding tube. It is a short tube that is inserted through the skin into a vein. A small needle is used to insert this catheter. The needle is removed, and the catheter stays in the vein. A PIV can be placed in the arm, hand, leg, foot and sometimes in the scalp. There may be some discomfort as the needle and catheter are inserted. We try to keep your child as comfortable as possible during the placement. We can provide options for increasing comfort for your child such as positioning, distraction, guided imagery and medicines, depending on your child's age and medical condition. Child Life Specialists may be available to help your child cope with PIV placement. A dressing is placed over the PIV when it is inserted. The dressing needs to be changed by a nurse if it is wet, soiled or bloody. It will also be changed if it becomes loose or begins to fall off. Hourly PIV site checks:
For safety reasons, we will be looking at the PIV every hour while fluids are going through it, even when your child is asleep. The nurse will need to check the PIV and dressing by turning on a light and comparing both arms or legs. We may need to reposition your child and remove pajamas or anything blocking the PIV site. This allows us to have a full view of the extremity that the PIV is in. We check the site to keep your child safe from an IV infiltrate. An infiltrate is when IV fluid or medicine flow under the skin, rather than into the vein. This can irritate your child's skin. You can help partner with us to prevent infiltrates. Let us know if you see any redness, leaking or swelling. Also tell us if your child seems irritable or if he has any discomfort at his PIV site. Instructions for bathing:

Your child may shower or bathe with the dressing in place, but we need to protect the dressing.

**Patient instruction text sample 2**

Joint Aspiration

What is joint aspiration?

Joint aspiration is a procedure to remove fluid from the space around a joint. It is done using a needle and syringe. This is often done to relieve swelling
or to get fluid for analysis to diagnose a joint disorder or problem. It may
be done with a local anesthetic to reduce pain. Needle inserted into joint, drawing out
fluid. Joint aspiration is most often done on the knee. But fluid can also be removed
from other joints. These include the hip, ankle, shoulder, elbow, and wrist
joints. Other related procedures may be used to help diagnose joint problems. These
include X-ray, bone scan, MRI, CT scan, arthroscopy, and arthrography. Why might I need a joint aspiration?

Joint aspiration may be done to diagnose and help treat joint problems. These
conditions may be diagnosed by testing the fluid:

Gout

Various types of arthritis

Joint infection

Joint aspiration can also be done to remove a large amount of fluid around a
joint. Sometimes bursitis (inflammation of the bursa) causes fluid to collect
near a joint. Removing the fluid will decrease the pressure. It can relieve
pain and improve movement of the joint. A medicine may be injected after
removal of the fluid. This is done to help treat tendonitis or bursitis. There may be other reasons for your healthcare provider to advise a joint
aspiration. What are the risks of a joint aspiration?

All procedures have risks. The risks of this procedure include:

Discomfort at the aspiration site

Bruising at the aspiration site

Swelling at the aspiration site

Infection at the aspiration site

There may be other risks depending on your overall health. Talk about any
concerns with your healthcare provider before the procedure. How do I get ready for a joint aspiration?

**Patient instruction text sample 3**

Your healthcare provider will explain the procedure to you. Ask any questions that you have about the procedure. You may be asked to sign a consent form. This gives your permission to do the procedure. Read the form carefully. Ask questions if something is not clear. Tell your healthcare provider if you are sensitive to or are allergic to any medicines, latex, tape, and anesthetic medicines (local and general).

These instructions are for Children's Hospital of Philadelphia (CHOP) patients
to record their diet for 3 days so that it can be evaluated by a CHOP
Registered Dietician.

Instructions for completing a 3 Day Food/Intake Record:

To help us evaluate your child's diet, record everything your child eats and drinks for 3 full days including foods, beverages, nutritional supplements, shakes, and vitamins.Use food labels to give name brands of foods and to help with serving sizes.
Include the following information:
Time of day: list the time of day the food or beverage was consumed.
Description of foods: list all foods, drinks, snacks, food extras (butter, oil, margarine, and salad dressing), vitamins, minerals, or other supplements (examples: Fish Oil, DHA).
Be specific!Include the brand name and how the food was prepared (fried, baked, breaded).Add important details such as: milk (1%, 2%, whole); instant oatmeal (original, lower-sugar flavored pack); ranch dressing (low-fat, fat-free).
Include the name of all fast food or chain restaurants.Tell us the menu item and the size (examples: kid's meal, 1/4 pound, small, etc.).
Amount Consumed: list how much food or beverage was consumed, not the amount served.

Tips for measuring

Food Scale Food scale should be used for specific weights of any food.
Measuring spoons should be used for:
Jelly/jam
Butter/margarine
Cream cheese
Measuring Spoons Oils
Sugar/salt
Ketchup
Salad dressing
Gravy/sauce
Liquid Measuring Cup Liquid measuring cups should be used for:
Water
Milk
Juices
Other beverages
Measuring Cups Dry measuring cups should be used for:
Rice/beans
Vegetables/fruit
Snacks
Nuts
Pretzels
Baby food
Pasta/cereal
Food labels should be used to look at serving size and specific amounts in ounces (oz.), grams (g), or milliliters (mL).
nutrition label facts

See a sample intake record and complete your child's
record.

**Simple Wikipedia text sample 1**

Ebola, pronounced E- Bo-la, is a hemorrhagic fever virus. (Hemorrhagic means causes very heavy bleeding. )It is a very deadly disease. 50-90% of people who get Ebola die. People who get infected with Ebola virus get the disease Ebola hemorrhagic fever. How people get EbolaIn people, transmission (giving the virus to someone) happens because of touching fluids with the virus. People with Ebola hemorrhagic (bleeding) fever bleed very much. They have blood in diarrhea and vomit. They bleed from their noses, mouths, and genitals. These liquids are very infectious (they can make other people get the virus. )Symptoms of Ebola When people get Ebola the first symptoms look like some other diseases. People get fever, feel very tired, have headache (pain in the head),pain in the stomach, pain in joints, and pain in the throat. Sometimes people think they have other diseases like malaria or typhoid fever. Later, people get much more sick. They have very bad bleeding. They get shock: low blood pressure, fast pulse (heart rate), and low blood circulation to the body. This causes organs to get very sick. The organs stop working. This is called organ failure. Then, even if they make it to a hospital, most die. Ebola cannot be caught through the air. Treatment of Ebola There is no special treatment for Ebola hemorrhagic fever. But if people get care from doctors and nurses more live. This is called supportive care. Supportive care can be fluids and blood given in people's veins. It can be medicines to make their blood pressure and blood circulation better. When people in a place get Ebola, it is called an outbreak. If doctors see an outbreak is happening, they try to stop it. They stop it by isolating people with infection. This means keeping the blood and fluids from people with Ebola from touching other people. Then other people do not get the virus. When an outbreak happens, many people come to try and help stop it.

**Simple Wikipedia text sample 2**

Iron (Fe) is the second most common metal on Earth, and the most widely-used metal. It is element 26, a transition metal in Group 8. Its symbol is Fe, from the Latin word for iron, ferrum. Its atomic number is 26 and its mass number is 55. 85. It is used a lot because it is very strong and cheap. Iron is the main ingredient used to make steel. Raw iron is attracted to magnets, but it is not actually a magnet, however it can be used to make an electromagnet. Iron is a grey, silvery metal. It is magnetic. It is easy to mine and make, which is why it is so useful. Pure iron is soft and very malleable and is able to stretch a lot, while steel (iron mixed with a little carbon) is stronger and does not stretch as much as iron. Iron is reactive. It reacts with most acids like sulfuric acid. It makes ferrous sulfate when reacted with sulfuric acid. This reaction with sulfuric acid is used to clean metal. Iron reacts with air and water to make rust. When the rust flakes off, more iron is exposed allowing more iron to rust. Eventually, the whole piece of iron is rusted away. Other metals like aluminum do not rust away. Iron can be alloyed with chromium and carbon to make stainless steel, which does not rust under most conditions. Iron powder can react with sulfur to make iron(II) sulfide, a hard black solid. Iron also reacts with the halogens to make iron(III) halides, like iron(III) chloride. Iron reacts with the hydrohalic acids to make iron(II) halides like iron(II) chloride. Iron makes chemical compounds with other elements. Normally the other element oxidizes iron. Sometimes two electrons are taken and sometimes three. Compounds where iron has two electrons taken are called ferrous compounds. Compounds where iron has three electrons taken are called ferric compounds. Ferrous compounds have iron in its +2 oxidation state. Ferric compounds have iron in its +3 oxidation state. Iron compounds can be black, brown, yellow, green, or purple.

**Simple Wikipedia text sample 3**

Pressure means how much something is pushing on something else. It is expressed as force per unit area:P=F/AIn technology, pressure is often specified in multiples of atmospheric pressure. It can also be defined as the thrust acting per unit area. Since pressure depends on both force and area, the same amount of force applied by a hand will make less pressure than when applied by something smaller, like a finger. Pressure is also related to density. By the ideal gas law, for example, doubling the pressure can put twice as much gas in a bottle. To measure the amount of pressure people use these units:

**WebMD text sample 1**

Editor's Note: For the latest updates on the
2020coronavirus outbreak, see our
news
coverage.

Coronavirus is here and it seems like the world is living out the movie
“Contagion.”As a doctor and a mom, I’m being asked a lot of questions.Here
are my top 5:

Why are so many events being cancelled?Should I stay home from work?

The short answer is that viruses spread through contact with people.If there
is a large event with many people attending, shaking hands and being in close
quarters, infections are likely to spread quickly.This is what leads to
“exponential” cases.And here’s the truth:Our hospitals do not have enough
beds to handle millions of
cases, especially those that are critically ill.And this is
a true danger for all of us.To help prevent this from happening, we need to
cancel events, do social distancing and yes, even work remotely.

Should I travel or should I cancel my plans?

Travel can expose you to viruses.COVID-19 can live on surfaces for over 24
hours, according to some studies and some airplanes are not wiped down after
every flight.If you do choose to travel, make sure you have disinfecting
wipes to clean where you are sitting – get the arm rests, the touch screen if
there is one and the food tray -- and wash your hands well after the flight.
Lastly, as for now, avoid international travel as there are many restrictions.
If you really need to get away, maybe a road trip to a close location is a
better option.

What about grandma?Can we see her?

People over the age of 60 are more at risk for serious problems like breathing
issues, pneumonia and even death.If you are exposed to coronavirus or any
viruses, you may want to stay away away from the elderly for about at least
two weeks, even if you don’t feel sick.Even if you aren’t exposed to cough or
viruses, you could be inadvertently a carrier, so wash your hands and keep
your distance from anyone older or anyone whose immune system could be down.

**WebMD text sample 2**

I was a bit worried that something didn’t seem right when I examined Kassi, a
5-month-old baby, who had been brought into the ER with a broken arm.Her
parents seemed wonderful, and they were very co-operative, but their story
that she had simply “fallen wrong” from a sitting position didn’t make sense.
I did more digging and learned that a recent job loss had increased the stress
level in the home dramatically – tensions were running high.Ultimately, we
learned that Kassi’s arm broke when her Dad, who was very stressed out from
his job loss, yanked her arm too hard one day when Kassi wouldn’t stop crying.

This story has been running through my mind as the
COVID-19 pandemic has been unfolding.We
know that an increase in financial, relational, and health-related stress
increases the risk for child abuse.And COVID-19 has brought unprecedented
levels of stress for all of us.For some, it’s adjusting to being constantly
at home, trying to work from home while homeschooling children, or being older
and isolated due to fears of getting the infection.For others, the situation
is even worse – job loss, financial uncertainty, living in crowded situations,
or within a bad relationship.This pressure cooker can inevitably lead to
lashing out on the most vulnerable, the children in the household.

So what can we do to help these stressed out families where kids may be at
risk?If you know of a family who may be under stress from job loss or other
pressures, here are a few suggestions:

If you suspect a child is getting abused, reach out to authorities.A child
who is subject to violence may get withdrawn and not want to talk, they may
have injuries that don’t make sense, or may not want to come out of their
home.With social distancing, what you see and hear from a troubled child may
be limited, but if you are worried, call the police or Childhelp National
Child Abuse Hotline (1-800-4AChild).They
will know what to do to make sure that the child is safe.

**WebMD text sample 3**

Drug development is an expensive, time-consuming, and often unsuccessful
endeavor — developing new drugs can often take more than 10 years, cost more
than $2 billion, and fail nearly 90% of the time.And these numbers have not
been improving, which is especially worrisome for people suffering from life-
threatening diseases and patients with chronic conditions for which there are
no good therapies or treatments.Researchers hope artificial intelligence (AI)
can help speed up the process, create greater efficiency, and make the process
less expensive.Take a look at four ways AI could do that.

AI can find new and targeted drugs more quickly.The discovery of new
drugs is about predictions — that is, how well a drug may work for a specific
disease and how individual persons who have the disease might benefit.AI can
help fine-tune these predictions.AI might also help anticipate how new drugs,
especially new drug structures, might function.And since drugs are often used
in combination — most people take more than one medicine — learning how a new
drug and existing drugs might interact is critical for researchers to see if
it is worthwhile to pursue various phases of clinical trials.

AI can use the power of computing to tap into large databases.AI can look
at associations and patterns more effectively than human researchers to find
new indications for existing drugs.For example, the drug Viagra was being
studied for hypertension and heart disease when researchers noticed men who
were taking it were having erections.In another example, researchers noticed
that the drug Latisse, studied to treat high eye pressure and glaucoma, also
caused eye lashes to grow and become thicker.AI could help find those
patterns sooner so that researchers don’t have to rely on chance.

AI can improve clinical trial design, recruitment, and participation.Drug
studies have large dropout rates and often don’t enroll enough women and
minorities.Some trials never get started or completed because researchers
lack enough participants.AI can help identify the reasons for dropout or lack
of enrollment.That information could help refine a clinical trial’s process
and perhaps require fewer participants.
